# Development and Use of a Cardiac Clinical Guideline Mobile App in Australia: Acceptability and Multi-Methods Study

**DOI:** 10.2196/35599

**Published:** 2022-08-05

**Authors:** Stacey Matthews, Brooke Atkins, Natalie Walton, Julie-Anne Mitchell, Garry Jennings, Amanda K Buttery

**Affiliations:** 1 National Heart Foundation of Australia Melbourne Australia; 2 Royal Melbourne Hospital Cardiothoracic Surgery Unit Parkville Australia; 3 Baker Heart and Diabetes Institute Melbourne Australia

**Keywords:** mHealth, mobile heath, apps, app, guideline, cardiovascular disease, atrial fibrillation, heart failure, heart, cardiac, cardiovascular, acute coronary syndrome, smartphone, implementation, digital health, develop, evaluate, evaluation, Australia

## Abstract

**Background:**

Implementation of clinical guidelines into routine practice remains highly variable. Strategies to increase guideline uptake include developing digital tools and mobile apps for use in clinical practice. The National Heart Foundation of Australia in collaboration with the Cardiac Society of Australia and New Zealand published 3 key cardiac clinical guidelines, including the Australian clinical guidelines for the (1) prevention and detection of atrial fibrillation, (2) detection and management of heart failure, and (3) management of acute coronary syndromes. To improve access and uptake for health care providers, we developed the Smart Heart Guideline App.

**Objective:**

This study aims to evaluate the acceptability, implementation, and usability of an Australian-specific cardiac guidelines mobile app.

**Methods:**

We used an iterative multiple methods development and implementation approach. First, we conducted a cross-sectional web-based survey with end users (n=504 health professionals) in 2017 to determine the acceptability of an Australian-specific cardiac clinical guidelines mobile app. Second, the Smart Heart Guidelines app was created using a design, user testing, and revision process. The app includes interactive algorithms and flowcharts to inform diagnosis and management at the point of care. The freely available app was launched in October 2019 on iOS and Android operating systems and promoted and implemented using multiple methods. Third, data from 2 annual national cross-sectional general practitioner (GP) surveys in 2019 and 2020 were evaluated to understand the awareness and use of the clinical guidelines and the app. Fourth, data from the app stores were analyzed between October 1, 2019, and June 30, 2021, to evaluate usage.

**Results:**

Most health professionals surveyed (447/504, 89%) reported accessing resources electronically, and most (318/504, 63%) reported that they would use an Australian-specific cardiac guidelines app. GPs surveyed in 2019 were aware of the heart failure (159/312, 51%) and atrial fibrillation (140/312, 45%) guidelines, and in 2020, a total of 34 of 189 (18%) reported that they were aware of the app. The app was downloaded 11,313 times (7483, 66% from the Apple App Store; 3830, 34% from Google Play) during the first 20-month period. Most downloads (6300/7483, 84%) were a result of searching for the app in the stores. Monthly download rates varied. App Store data showed that people used the app twice (on average 2.06 times) during the 20 months. Many (3256/3830, 85%) Android users deleted the app.

**Conclusions:**

Health professionals supported the development of the Smart Heart Guidelines app. Although initial downloads were promising, the frequency of using the app was low and deletion rates were high. Further evaluation of users’ experience of the most and least useful components of the app is needed.

## Introduction

Cardiovascular disease is the leading cause of death in Australia and a nationwide priority for research [1]. National clinical guidelines have been developed to inform decision-making among health professionals in the diagnosis and management of cardiovascular disease. Implementation of clinical guidelines can prevent avoidable harm, improve resource use, and reduce variation in care [2]. Despite this, implementation of clinical guidelines into routine practice remains highly variable, suboptimal, and concerningly low in some areas of health care [3]. In Australia, the National Health and Medical Research Council advises improving access and uptake of clinical guidelines by presenting content in multiple formats that are tailored to users’ needs [2].

Smartphone apps are a convenient way for health professionals to access health information in a timely manner. In recent years, international organizations including the European Society of Cardiology and the American Heart Association have developed mobile apps providing digital access to their guidelines and specific apps for interactive decision support tools for real-time use in clinical practice and cardiovascular risk calculation [4,5]. Similarly, other health disciplines including anesthesiology, pediatrics, and dermatology have developed apps to improve access and adherence to guideline recommendations in clinical practice [5-8]. With the advent of “living guidelines” and its methods of continuous evidence surveillance and recommendation updates, digital authoring, and web-based publication platforms such as Making Grade the Irresistible Choice (MAGICapp) have been developed, publishing over 190 living guidelines, including the Australian guidelines for care for people with COVID-19 [9]. These developments compliment a trend toward greater utilization of digital platforms and smart phone use by doctors in Australia [10,11].

The National Heart Foundation of Australia (Heart Foundation) is an independent, not-for-profit organization that funds cardiovascular research and, in partnership with the Cardiac Society of Australia and New Zealand and other organizations, publishes clinical practice guidelines and position statements in areas where guidance will have the biggest impact.

The study aim was to evaluate the acceptability, implementation, and usage of an Australian-specific mobile app to improve the awareness and use of 3 Australian cardiac clinical guidelines for the (1) prevention and detection of atrial fibrillation 2018, (2) detection and management of heart failure 2018, and (3) Australian clinical guidelines for the management of acute coronary syndromes 2016 [12-14].

## Methods

### Overview

We used an iterative multiple methods approach, drawing on established methods in mobile app development, implementation, and evaluation [15]. First, we conducted a cross-sectional web-based survey of end-users to determine the acceptability of an Australian-specific mobile app to access cardiac clinical guidelines. Second, the Smart Heart Guidelines app was developed and launched on Google Play and Apple App Store (App Store) and promoted using multiple methods. Third, the app was promoted and implemented. Fourth, data from 2 annual cross-sectional national general practitioner (GP) surveys in 2019 and 2020 were evaluated to assess awareness and use of the clinical guidelines and the app. Fifth, data from the app stores were collated to evaluate app use. Each of these steps are now described.

### Health Professional Acceptability Survey

A web-based survey designed for health professionals likely to use a cardiology guidelines app was developed and distributed in 2017. The survey contained 10 questions including the frequency of searching for information about the prevention or management of cardiovascular disease, methods of searching, and the likelihood of using an Australian-specific mobile app to access cardiac clinical guidelines. The survey was distributed through the Heart Foundation’s social media accounts and promotion in the organization’s monthly newsletter (the mailing list comprised of approximately 15000 health professionals: average open rates of 21%, 3150/15000). Survey responses informed the development of the app.

### Development of the Smart Heart Guidelines App

The Heart Foundation collaborated with a global app developer, experienced with international cardiology apps in Europe and the United States. The app was created in iOS (Apple) and Android formats.

Three clinical guidelines were converted from publication in a PDF format in a peer-reviewed journal into the app. The app included the guidelines for acute coronary syndromes, atrial fibrillation, and heart failure [12-14]. The app was organized into 3 sections for each clinical guidelines and contained a main page, table of contents, interactive tools and algorithms (to support clinical decision-making), and a table of key recommendations, as shown in Multimedia Appendix 1. The app was created using a design, user testing, and revision process.

The app was registered as a class 1 software–based medical device with the Australian Register of Therapeutic Goods in 2019, in accordance with national legislation [16]. Registration requirements include ongoing monitoring of safety, quality, and performance. Users had to declare that they were a health professional to download the app. The freely available (no cost) Smart Heart Guidelines app was launched on both the App Store and Google Play in October 2019.

### Promoting and Implementing the App

Implementation strategies used to promote the app included printed and electronic flyers, containing a quick response code distributed at health professional educational meetings and events. Direct email and newsletters to multiple health professional groups and paid advertising in health professional journals was used. Promotion of the app on multiple webpages associated with the clinical guidelines was also undertaken.

### Annual Cross-sectional Awareness Surveys to General Practitioners in 2019 and 2020

The Heart Foundation develops and distributes a nationwide cross-sectional survey to GPs annually since 2010. These surveys collect feedback about views, attitudes, awareness and the use of Heart Foundation resources and clinical guidelines. The 2019 and 2020 surveys were distributed to a sample of approximately 4000 GPs identified from the Medical Directory of Australia [17]. The 2019 annual survey contained 24 items and was distributed and open between October and November 2019. GPs could respond on the web, using the software platform Typeform or via a paper version [18]. In 2020, the 21-item survey was only available on the web and was open from October 2020 to November 2020 [18]. Both surveys contained a combination of closed-ended questions and Likert rating scales.

### Evaluation of App Usage From the App Store and Google Play

Usage data for the app was retrieved from the App Store and Google Play during the period between October 1, 2019, and June 30, 2021. Data from both app stores showed the total number of downloads of the app and the conversion rate (number of downloads divided by the number of impressions) during this period. Data from Google Play showed the retention rate (percentage of users who had not uninstalled the app from their device). The App Store report provided the number of impressions (the number of times an app appears in an App Store search), product page views (the number of times a user viewed the apps product page in the App Store), and average use (number of sessions per device divided by the total number of users).

### Ethical Considerations

Heart Foundation surveys are approved through routine governance organizational processes. Data collected from the surveys were anonymized and informed consent was assumed at the time of survey participation. Therefore, there was no ethical application made for this multi-methods study.

### Data Handling and Statistical Analyses

Descriptive statistics were used to summarize the health professional acceptability survey from 2017 and the GP surveys in 2019 and 2020. Google Analytics was used to evaluate app data from the App Store and Google Play. 

## Results

### Health Professional Acceptability Survey in 2017

There were 504 respondents, from all 6 states and 2 territories in Australia. Most were nurses (198/504, 39%), allied health professionals (132/504, 26%), GPs and cardiologists (45/504, 9%), researchers (11/504, 2%), or identified as other (118/504, 23%). Respondents were from a variety of disciplines including public health (131/504, 26%), private practice (121/504, 24%), community health (86/504, 17%), research institute (55/504, 11%), private hospital (35/504, 7%), health promotion (25/504, 5%), and others (50/504, 10%). Most (447/504, 89%) reported accessing the Heart Foundation’s resources electronically. Most health professionals reported using Microsoft software (246/504, 63%) Apple iOS (162/504, 32%), or Android devices (63/504, 13%). The majority of health professionals (369/504, 73%) reported they would use a mobile device (a tablet or mobile phone) to access health professional resources, although the majority (465/504, 92%) currently did not. The majority (314/504, 62%) reported that they would be likely or very likely to use an Australian-specific mobile app to access the clinical guidelines and resources.

### Promotion and Implementation the App

Implementation and promotional strategies for the app are summarized in Table 1. Promotion strategies had a potential reach to over 73,000 individuals.

**Table 1:** Methods used to promote and implement the Smart Heart Guidelines app.

Method			Dates promoted		Recipients
**Internal**					
	Direct email to NHFA^a^ staff	November 22, 2019		90 staff members	
	NHFA all staff newsletter	November 27, 2019		272 staff members	
**External**					
	NHFA webpage on the Smart Heart Guidelines app	Live from November 11, 2019		6721 page views (November 11, 2019 to June 30, 2021)	
	NHFA newsletter to health care professionals	Monthly promotion between November 2019 and September 2020		Approximately 20,000 heath care professionals subscribed	
	Direct email to health organizations (eg, Stroke Foundation)	November 11, 2019		69 unique IP addresses	
	NHFA advisory committees and guideline writing groups	November 22, 2019		40 members	
	Promoted in the Australian Primary Health Care Nurses Association newsletter	November 2019		Approximately 3500 members	
	Direct email to the CSANZ^b^	December 2019		Approximately 3000 members	
	Print the advertisement in the RACGP^c^ newsletter	December 1, 2019		Approximately 40,000 general practitioners	
	Society page in *Heart, Lung, Circulation*	February 2020		>2000 cardiologist and cardiac surgeons in Australia	

^a^NFHA: National Heart Foundation of Australia.

^b^CSANZ: Cardiac Society of Australia and New Zealand.

^c^RACGP: Royal Australian College of General Practitioners.

### Annual Cross-sectional Awareness Surveys to General Practitioners in 2019 and 2020

From the 4000 GPs on the distribution list, 312 GPs responded in 2019, and 189 GPs responded in 2020. Respondents’ awareness of specific cardiac clinical guidelines and reported the use of guidelines and the Smart Heart Guideline app are presented in Table 2. 

**Table 2:** Frequencies of general practitioners (GPs) who reported being aware and frequently using specific guideline resources in the annual GP surveys in 2019 (n=312) and 2020 (n=189).

Surveys			Heart failure^a^, n (%)		Atrial fibrillation^b^, n (%)		App^c^, n (%)
**GP survey 2019**							
	Aware of the resource	159 (51)		140 (45)		Not asked	
	Frequently uses the resource	9 (7)		19 (6)		Not asked	
**GP survey 2020**							
	Aware of the resource	113 (60)		102 (54)		34 (18)	
	Frequently uses the resource	11 (6)		11(6)		10 (5)	

^a^Guidelines for the prevention, detection, and management of heart failure in Australia 2018 [13].

^b^Guidelines for the prevention and management of atrial fibrillation in Australia 2018 [12].

^c^Smart Heart Guideline app.

### Data From the App Store and Google Play

During the period from October 1, 2019, to June 30, 2021, the Smart Heart Guideline app had 11,313 downloads (n=7483, 66% from the App Store; n=3830, 34% from Google Play). Data from the App Store indicated over 59,900 impressions (the number of times the Smart Heart Guideline App appeared in an App Store search) with 9000 product page views (number of times a user viewed the apps product page in the App Store), resulting in 7483 downloads. Of these downloads, the majority (6300/7483, 84%) were a direct result of searching for the Smart Heart Guideline app in the App Store. The remaining downloads resulted from accessing the app’s product page via the organization’s webpage (610/7483, 8%) from browsing the App Store (n=346, 5%) or from an app referrer (a link within another app; n=214, 3%). The app had consistent conversion rates (number of downloads divided by the number of impressions) throughout this period in both the App Store and Google Play. Data from the App Store showed that most downloads were on a mobile device (6951/7483, 93%) with fewer downloads on a tablet (532/7483, 7%). The App Store data showed low average use of the app, with an average of 2.06 sessions per user during the 20-month period. Additionally, Google Play data showed that from the 3830 downloads, most (3260/3830, 85%) resulted in user losses (app deletion by users). Download data from the App stores are presented in Multimedia Appendix 2.

## Discussion

### Principal Findings

Following a survey among end users, which revealed that approximately two-thirds (318/504, 63%) of health professional reported being “likely” or “very likely” to use an Australian-specific mobile app to access cardiology guidelines, the Smart Heart Guidelines app was launched in October 2019. Downloads of the app during the first 20 months from users deliberately searching for the Smart Heart Guideline app in the App Store and Google Play indicate that promotional and implementation activities were useful in raising awareness of the app. The total number of app downloads (>11,000), primarily in smartphones in the first 20 months from app launch, is encouraging. However, usage data show people tended to use the app only a couple of times and many deleted the app, indicating it may not be meeting users’ needs. Responses from the 2019 and 2020 GP surveys demonstrate a consistent lack of awareness and use of clinical guidelines, despite efforts to disseminate broadly and improve accessibility, including open access publishing. Although 18% (34/189) of GPs reported awareness of the Smart Heart Guidelines app in the 2020 GP survey, fewer (10/189, 5%) reported frequently using it.

### Comparisons With Prior Work

Guideline implementation is notoriously challenging [3]. Internationally, there have been mixed experiences with the development and use of clinician-facing guideline apps. The National Institute for Health and Care Excellence, United Kingdom, a major developer of clinical guidelines, launched a guideline app in 2012 only to retire it in 2018 because app use stagnated and more people were directly accessing their website for information [19]. It is important to consider the purposes of a guideline app, and if it is created for voluntary download and passive access to guideline information, it may not be meaningful to clinicians and may have low impact on practice and behavioral change. Contrastingly, apps involving more interactive and specific decision support tools may be better in improving guideline adherence and changing patient outcomes. The European Society of Cardiology Atrial Fibrillation clinical guideline app has demonstrated the value of integrating novel digital technology into clinical practice, with potential for optimizing health care professionals’ adherence to recommendations of pharmacological and interventional therapy for patients with atrial fibrillation [4]. Our App included a combination of guideline information and interactive decision support tools.

In a literature review of medical smartphone apps in clinical decision support, Watson et al [20] identified 48 trials and one Cochrane review finding that while diagnostic accuracy studies are plentiful, studies to determine whether guideline apps improve adherence to guidelines are only beginning to emerge, usually in the form of before-and-after studies, often in global health. For example, in India, in a study of over 6000 participants, a nurse‐facilitated smartphone‐enabled hypertension and diabetes mellitus intervention in primary care was associated with significant improvements in blood pressure and blood glucose control over 18 months [21]. The nurse examined and entered patient parameters into a mobile phone–based clinical decision support system to generate a prescription, which was reviewed by a physician [21]. In contrast, in a small randomized controlled trial of pediatric doctors in the United States in a hospital simulation study, a guideline app developed to support and drive cardiopulmonary resuscitation in real time improved guideline adherence compared with other tools including pediatric advanced life support pocket cards. The app was associated with a shorter time to first and subsequent defibrillation attempts and fewer medication errors.

A reported barrier to health professionals’ uptake of guideline apps is the lack of an international regulatory framework to ensure that the apps are evidence-based and held to a quality standard [15,22]. In Australia, therapeutic goods including medical software must be entered in the Australian Register of Therapeutic Goods, and registration of the app as a class 1 medical device may have positively influenced the uptake of the app. Studies investigating if the investment in creating and maintaining a guideline app is superior to other guideline implementation strategies are lacking [20,23]. It was beyond the scope of this study to undertake a cost-benefit analysis of the app; however, reviews of cost-effectiveness of digital health interventions in the management of cardiovascular disease are generally promising [24].

### Strengths and Limitations

A strength of this study is the use of multiple methods used to identify the acceptability of an Australian-specific cardiology clinical guidelines app and comparing App Store and Google Play usage data and GP experience survey data over 2 years (2019 and 2020). The app included both guideline information along with clinical decision support tools and interactive algorithms. However, this study has limitations. First, although the acceptability survey had over 500 respondents, only a small proportion of them were GPs and cardiologists, the main intended users of the app, potentially limiting the representativeness of this sample. Second, response rates to the 2019 and 2020 surveys were low, limiting the generalizability of the results. Third, comparisons between the app’s use across the App Store and Google Play was restricted as data capture is not consistent across these commercial platforms. Fourth, we were unable to comment on health professional behavior change resulting from app use, as it was beyond the scope of this study, and alternate methods are required to understand how access to the app influenced clinical practice healthcare service delivery.

### Future Directions

This formative research provides a summary of why and how an Australian-specific cardiac guideline app was developed, launched, promoted, and initially used. Future research could involve qualitative interviews with app users to explore which aspects of the app they find most and least useful and to discover any unmet needs of health professionals using the app. More tailored research into the use of clinical decision aids and support tools in the app would help better understand whether the app changes health professional behaviors and improves patient outcomes.

### Conclusions

The development of the Smart Heart Guideline app, a cardiology-specific guideline app in Australia, was indicated from surveying health professionals. The app incorporated guideline information and clinical support decision aids. Although downloads of the app from among >11,000 users in the first 20 months was a promising finding, the frequency of using the app was low and deletion of the app was high. Further evaluation of the app is needed to understand the most and least useful aspects and to understand if using the app improves guideline adherence and impacts patient outcomes.

## Appendix

Multimedia Appendix 1 Smart Heart App user interface.

Multimedia Appendix 2 Number of Smart Heart guidelines app downloads by month from the Apple app store and Google Play store.

## Abbreviations

GP: general practitioner
