# Supplementary figures and images for: Development and Use of a Cardiac Clinical Guideline Mobile App in Australia: Acceptability and Multi-Methods Study
Source: JMIR Form Res. 2022 Aug 5;6(8):e35599. doi: 10.2196/35599 (PMC9391980; doi:10.2196/35599)

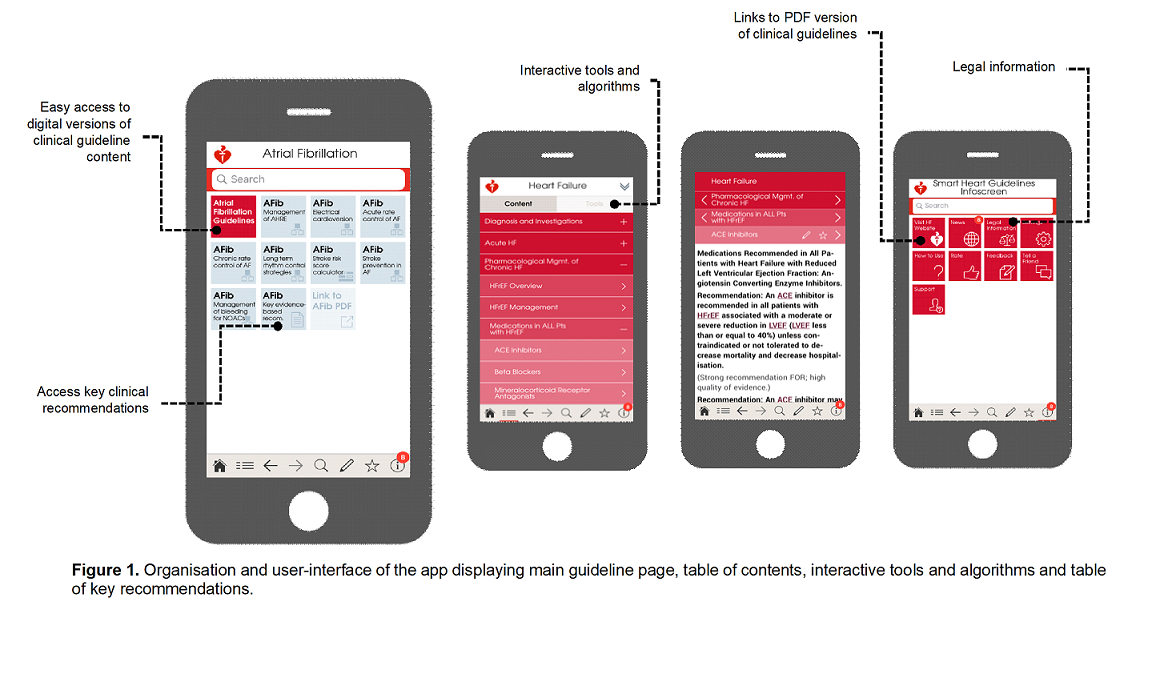

Supplement: Multimedia Appendix 1 [file formative_v6i8e35599_app1.png]

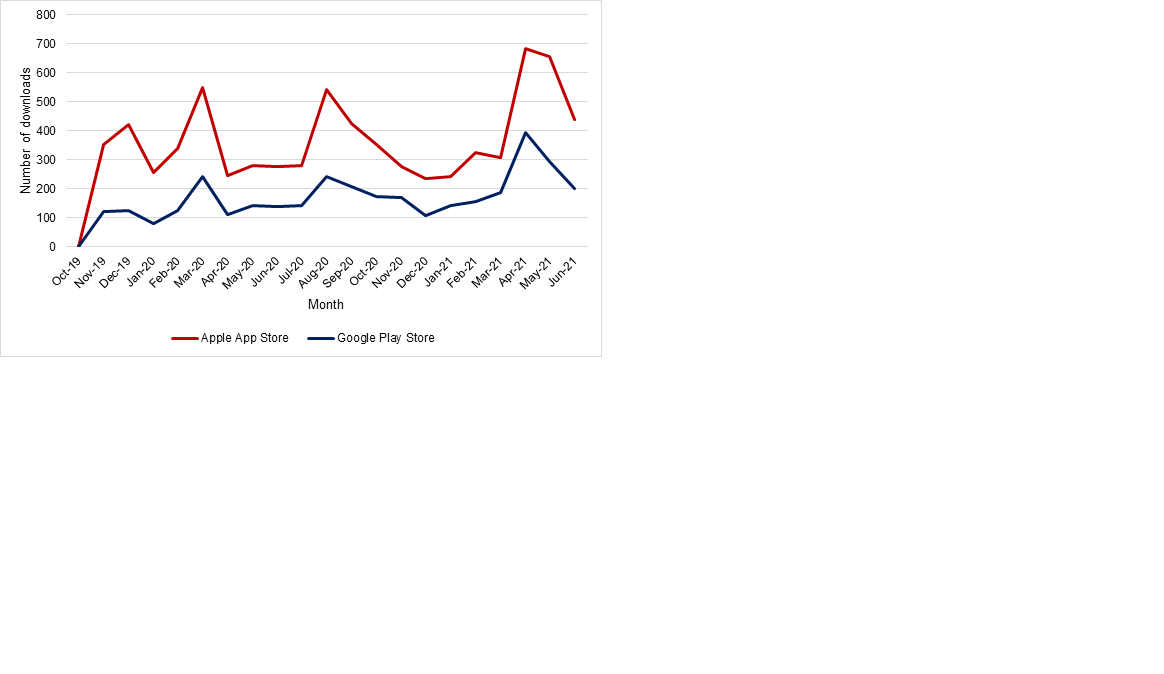

Supplement: Multimedia Appendix 2 [file formative_v6i8e35599_app2.png]
